# Bacteraemia Associated with *Bilophila wadsworthia*: A Rare Case Presentation from Hungary

**DOI:** 10.3390/pathogens13090749

**Published:** 2024-08-31

**Authors:** Renátó Kovács, Tamás Árokszállási, Aliz Bozó, Ágnes Jakab, Krisztina Szonja Bábel, Máté Héja, Kitti Bernadett Kovács, Bence Balázs, Eszter Vitális, László Majoros, Zoltán Tóth

**Affiliations:** 1 Department of Medical Microbiology, Faculty of Medicine, University of Debrecen, 4032 Debrecen, Hungary; bozo.aliz@med.unideb.hu (A.B.); jakab.agnes@med.unideb.hu (Á.J.); balazs.bence@med.unideb.hu (B.B.); major@med.unideb.hu (L.M.); toth.zoltan@med.unideb.hu (Z.T.); 2 Medical Microbiology, Clinical Centre, University of Debrecen, 4032 Debrecen, Hungary; 3 Department of Neurology, Faculty of Medicine, University of Debrecen, 4032 Debrecen, Hungary; arokszallasi.tamas@med.unideb.hu (T.Á.); babel.szonja@med.unideb.hu (K.S.B.); heja.mate@med.unideb.hu (M.H.); kovacs.kitti@med.unideb.hu (K.B.K.); 4 Infectology Clinic, Clinical Centre, University of Debrecen, 4032 Debrecen, Hungary; vitalis.eszter@med.unideb.hu

**Keywords:** *Bilophila wadsworthia*, anaerobic bacteraemia, whole-genome sequencing

## Abstract

*Bilophila wadsworthia* is a Gram-negative anaerobic bacterium. In current study, it was identified in the bloodstream of a 69-year-old man admitted to the Neurology Clinic at the University of Debrecen, Clinical Centre, Hungary, for internal carotid artery stent implantation. Bacteraemia caused by *B. wadsworthia* is extremely rare, with very few cases reported worldwide. This case is notable because it is the first instance in which whole-genome sequencing of *B. wadsworthia* derived from blood was performed. Moreover, the sequence data have been deposited in a public database.

## 1. Introduction

Anaerobic bacterial bloodstream infections remain a remarkable and underestimated condition in clinical practice [1,2]. Based on current epidemiological data, the proportion of anaerobic bacteraemia ranges from 0.5% to 15.0%. The mortality rate is relatively high, ranging from 15% to 45% depending on the causative agent [1,2]. The most frequently involved microorganisms in anaerobic bacteriaemia are *Bacteroides* spp., followed by *Fusobacterium* spp. and *Clostridium* spp. [1,2,3]. We recently encountered a case of clinically significant bacteraemia due to *Bilophila wadsworthia* isolated by pure culture.

*B. wadsworthia* is a Gram-negative, rod-shaped, asaccharolytic anaerobic bacterium belonging to the *Desulfovibrionaceae* family that exhibits strong catalase positivity [4]. Based on the limited number of clinical studies of *B. wadsworthia*, the preferred ecological niche of this species is the gastrointestinal tract. Nevertheless, it is occasionally isolated from the oral cavity and vaginal tract [4]. It is most commonly isolated from the large intestine and is present in more than half of all appendiceal specimens obtained from patients with appendicitis [4]. The widespread usage of matrix-assisted laser desorption–ionisation time-of-flight mass spectrometry (MALDI-TOF MS) has significantly improved the identification of various anaerobic bacteria [5]. Despite this, *B. wadsworthia* has been overlooked or misidentified because it exhibits slow growth on routine anaerobic blood agar plates, on which it forms small, nondescript, translucent colonies [4]. Based on available case reports, *B. wadsworthia* has been isolated from abscesses, axillary hidradenitis suppurativa, osteomyelitis, joint fluid, and pleural fluid [4,6,7,8]. To the best of our knowledge, however, only seven case reports of *B. wadsworthia* bacteraemia have been reported since 1989, which was the year in which this species was first isolated [9]. Two cases occurred in patients with liver abscesses, and one occurred as transient bacteraemia in a 66-year-old patient with abdominal distension and an aortic aneurysm [4,10]. Moreover, in 2022, Acker et al. [9] reported an uncommon case of polymicrobial anaerobic bacteraemia involving the simultaneous identification of *Bacteroides fragilis*, *Eggerthella lenta*, *Ruminococcus gnavus*, and *B. wadsworthia*. We herein report the first case of *B. wadsworthia* bacteraemia in which whole-genome sequencing was performed and made available in a public database.

## 2. Case Report

### 2.1. Medical History and Clinical Manifestations

**Figure 1:**
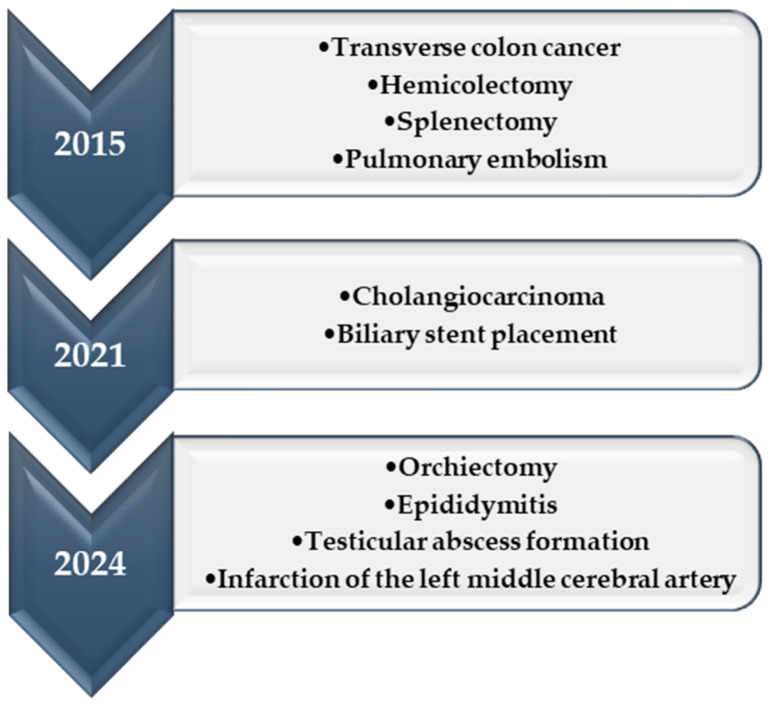
Patient medical history timeline.

In 2024, a 69-year-old man presented to the Neurology Clinic of the Clinical Centre at the University of Debrecen, Hungary, for internal carotid artery stent implantation. His medical history included diabetes mellitus, chronic pancreatitis, cholelithiasis, rectal fistula surgery, bilateral Warthin tumours of the parotid glands, and parotid surgery. In 2015, he underwent hemicolectomy and splenectomy due to transverse colon cancer, followed by postoperative chemotherapy during which he developed a pulmonary embolism. In 2021, he was diagnosed with cholangiocarcinoma and underwent biliary stent placement to manage acute obstructive jaundice. In 2024, simple orchiectomy was performed to treat orchitis, epididymitis, and abscess formation (Figure 1).

In 2024, he was treated on the neurology ward (Nyíregyházi Jósa András Tagkórház, Szabolcs-Szatmár-Bereg County Teaching Hospital, Nyíregyháza, Hungary) because of right limb paresis, which we considered possibly caused by territorial infarction of the left middle cerebral artery. Carotid artery duplex ultrasound revealed approximately 90% stenosis of the left internal carotid artery, which was confirmed by computed tomography angiography. As this stenosis could be the source of the stroke, a neurologist indicated carotid artery stent implantation, and then, an interventional radiology consultation supported that endovascular care of the stenosis was technically feasible. Therefore, this was performed at the Neurology Clinic of the Clinical Centre at the University of Debrecen.

### 2.2. Initial Assessment and Haematological and Radiological Investigations

Upon arrival, the patient had a blood pressure of 119/69 mmHg, a heart rate of 72 beats/min, and no fever. His heart sounds were clear and rhythmic with no murmur, and he had no icterus, cyanosis, or oedema. A systolic murmur was detected at the right carotid site along with a slightly tachycardic pulse. General examination revealed no notable neurological symptoms. A healed surgical scar was present on the abdomen, and a chronic fistula with brownish, malodourous discharge was present on the right side of the umbilicus. Abdominal ultrasound examination revealed a strengthened epigastric venous collateral pathway, suggesting increased portal pressure. The gallbladder was enlarged to approximately 11.9 × 4.0 cm and exhibited echo-dense areas corresponding to gallstones. The intrahepatic bile ducts were broad. The general physical examination was otherwise unremarkable, with no tenderness or discomfort upon abdominal palpation.

The haematological analysis revealed neutrophilia (17.12 × 10^9^/L; normal range: 1.9–7.7 × 10^9^/L), hypokalaemia (3.1 mmol/L; normal range: 3.5–5.3 mmol/L), and anaemia (3.13 × 10^12^/L; normal range: 4.7–6.1 × 10^12^/L), with decreased haemoglobin (93 g/L; normal range: 130–165 g/L) and haematocrit (0.27; normal range: 0.39–0.5) levels. The C-reactive protein level (57.54 mg/L; normal range: <5.2 mg/L) and procalcitonin level (11.4 μg/L; normal range: <0.5 μg/L) were elevated. The rest of the blood analysis, including the renal and hepatic function indices and the urinalysis, showed values within the reference ranges.

### 2.3. Microbiology Investigations

Blood samples were obtained for both aerobic and anaerobic haemocultures. The two sets of blood cultures obtained from peripheral veins at presentation were inoculated into Bact/Alert FA Plus and Bact/Alert FN Plus bottles (bioMérieux SA, Marcy-l’Étoile, France) and then incubated in a Bact/Alert 3D system (bioMérieux SA, Marcy-l’Étoile, France). After 72 h of incubation, both anaerobic blood culture bottles yielded positive results. Gram staining directly from positive bottles revealed the presence of Gram-negative rod-shaped bacteria that formed small transparent colonies on Schaedler agar after 48 h of incubation under anaerobic conditions. Species identification by MALDI-TOF MS (Bruker Daltonics, Bremen, Germany) confirmed the isolate as *B. wadsworthia* (log score: 2.24 and 2.31). Susceptibility testing with several antibiotics was carried out by determining the minimal inhibitory concentration (MIC) using E-test methods (bioMérieux SA, Marcy-l’Étoile, France). The E-test strips were used on *Brucella* blood agar and incubated under anaerobic conditions at 37 °C for 48 h. The obtained susceptibility results were interpreted based on the Clinical and Laboratory Standards Institute M100-ED34:2024 guidelines for anaerobes [11]. The isolate was resistant to penicillin G (MIC: 16 mg/L) and ampicillin (MIC: 16 mg/L). However, it was susceptible to clindamycin (MIC: 0.064 mg/L), metronidazole (MIC: 0.032 mg/L), imipenem (MIC: 1 mg/L), meropenem (MIC: 0.016 mg/L), ertapenem (MIC: 0.5 mg/L), and moxifloxacin (MIC: 0.5 mg/L). The MICs of linezolid and tigecycline were 1 mg/L and 0.016 mg/L, respectively.

### 2.4. Treatment and Outcome

On day 0, the patient was admitted to the Neurology Clinic for stent implantation. The intervention occurred without complications. On the first postoperative day, the inflammatory parameters were elevated, and the patient had a fever (38.7 °C). Empirical antibiotic treatment was initiated with intravenous ceftriaxone (1 × 2000 mg) while awaiting the culture results. The patient’s vital signs temporarily improved; however, on day 6, *B. wadsworthia* bacteraemia was confirmed. Based on the infectologist consultation, the administered ceftriaxone treatment was supplemented with intravenous clindamycin (3 × 900 mg) for 5 days. The patient’s general status continuously improved. On postoperative day 1, the C-reactive protein level was 57.54 mg/L (reference range: <5.2 mg/L), and it had further decreased by day 7 (28.44 mg/L; reference range: <5.2 mg/L) and day 11 (7.1 mg/L). The patient had a favourable hospital course and was discharged after a 12-day stay in the ward. At the time of writing this paper, follow-up appointments with oncology and abdominal surgery were required because of a scheduled surgical intervention.

### 2.5. Whole-Genome Sequencing of Bilophila wadsworthia Isolate

**Figure 2:**
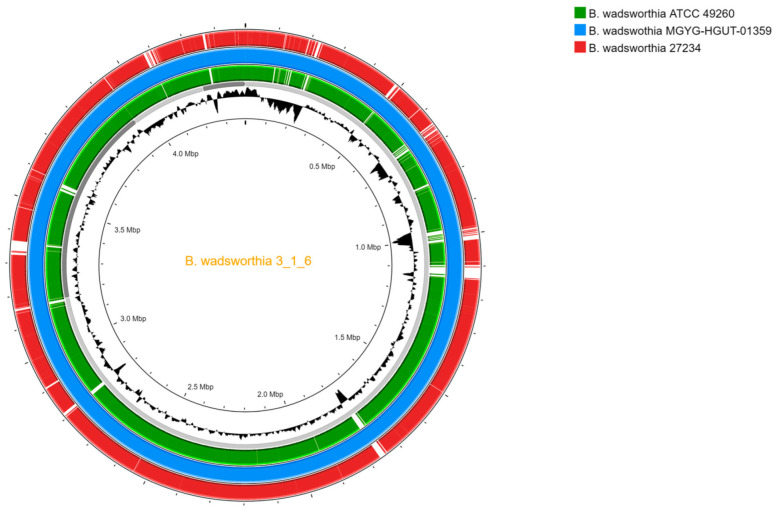
BLAST analysis results and visualization using ProkSEE in default mode. *B wadsworthia* 3_1_6 (Genbank acc. numb.: GCA_000185705.2) as reference. For comparative purposes, apart from the isolate presented in this study (*B. wadsworthia* 27234, Genbank acc. numb.: JBFRXO000000000), both *B. wadsworthia* ATCC 49260 (Genbank acc. numb.: GCA_000701705.1) and *B. wadsworthia* MGYG-HGUT-01359 (Genbank acc. numb.: GCA_902374275.1) are added to the analysis.

Nucleic acid isolation was performed with a QIAGEN DNeasy Blood and Tissue Kit (QIAGEN, Hilden, Germany) according to the manufacturer’s protocol regarding Gram-negative bacteria. Library preparation for Illumina sequencing was performed using an Illumina DNA-Prep library preparation kit (Illumina, San Diego, CA, USA), and short-read sequencing was performed on an Illumina MiSeq instrument with a target coverage of 80× in paired-end mode (2 × 150 bp). Library preparation and sequencing were performed at the Genomic Medicine and Bioinformatics Core Facility of the University of Debrecen, Hungary. For Oxford Nanopore Technologies (ONT) sequencing, the same isolated nucleic acid was used. The library was prepared with a Rapid Sequencing Kit V14 (SQKRAD114) and sequenced on a MinION instrument using an R10.4.1 flow cell (FLO-MIN114) with a target coverage of 500× (ONT, Oxford, UK). ONT base calling was performed using Dorado 7.3.11 with default parameters in the super accurate mode of MinKNOW software. Passed reads were assembled with Flye (with non-default parameters as follows: min_read_length: 10,000; flye_genome_size: 4,400,000; flye_asm_coverage: 50) and polished with Medaka as part of the Epi2Me Labs bacterial genome workflow [12,13]. The long-read assembly was polished with PolyPolish [14] using Illumina reads filtered with fastp [15] (parameters: --cut_front 20, --cut_tail 20, --cut_window_size 5, --cut_mean_quality 15, -q 15, -u 50, -f 18, -w 8, -l 95). Genome completeness was assessed using BUSCO v5 on gVolante with the odb_v10_Desulfovibrionaceae dataset and CheckM at KBase.us [16,17]. Similarity between the isolate and the two available reference genomes was evaluated using FastANI [18] and visualised with ProkSEE [19] BLAST results (Figure 2). Resistance determinants were screened using ResFinder [20].

## 3. Discussion

Anaerobic bacterial species are important aetiological agents in bloodstream infections. However, their prevalence and incidence are frequently underestimated because of their challenging isolation and fastidious nature [1,2,3]. Regarding the epidemiology of anaerobic bacteraemia, the probability of bloodstream infections caused by Gram-negative anaerobic bacteria is higher if the patient has a malignant disease, has undergone abdominal surgical intervention, or has received an organ transplant [2,3]. In these clinical scenarios, *Bacteroides* spp. are isolated most frequently, followed by *Fusobacterium* spp. and *Prevotella* spp. [1,2,3]. In the present case study, the patient had transverse colon cancer (cholangiocarcinoma), which predisposed him to Gram-negative anaerobic bacteraemia.

*Bilophila wadsworthia* was described in 1989 by Baron et al. [21] following its isolation from approximately half of anaerobic cultures of clinical specimens from patients with appendicitis. Based on current data, the number of *B. wadsworthia* bloodstream infections is very limited. These limited clinical data regarding *B. wadsworthia* bloodstream infections in the literature preclude detailed comparisons with our case. In one case, *B. wadsworthia* was associated with transient bacteraemia in a 66-year-old patient with abdominal distension [4]. In 1992, Kasten et al. [10] described a 43-year-old patient who was admitted to the intensive care unit for treatment of septic shock 1 month following a revascularisation procedure for mesenteric artery insufficiency. Two blood cultures were taken upon admission, from which *B. wadsworthia*, *Veillonella parvula*, and viridans streptococci were isolated [10]. In another case, a 62-year-old patient who had undergone subtotal gastrectomy, gastrojejunostomy, and chemotherapy was admitted to the hospital with a liver abscess. *Bacteroides fragilis*, *Bacteroides uniformis*, and *B. wadsworthia* were identified in one blood culture taken the day after admission, following 4 days of incubation [10]. Notably, the most likely source of the blood isolates in both cases was the hepatic abscess [10].

In the present case, the patient had undergone multiple gastrointestinal surgical interventions for the treatment of different cancers. Moreover, the physical examination revealed a fistula in his abdomen with brownish exudate. The presumed source of the transient *B. wadsworthia* bacteraemia in this patient was the unresolved abdominal wound infection. However, we could not culture *B. wadsworthia* from this wound; thus, the definitive source remained questionable. To the best of our knowledge, no genome studies on bloodstream infections have characterised *B. wadsworthia* at the genetic level. At the time of our analysis, genome sequences of only nine individual isolates of this species were publicly available. Furthermore, several datasets were derived from gastrointestinal tract-related metagenomic analyses. Nevertheless, because *B. wadsworthia* is a pathogenic bacterial species, the exploration of its genome-associated characteristics attracts great interest.

Although a large portion of the described *B. wadsworthia* isolates have been shown to be beta-lactamase producers, we were unable to identify such enzymes in the genome of our isolate despite the rather high MIC value of penicillin G. This may indicate that basic penicillins have intrinsically weak activity against this species regardless of their beta-lactamase status and should be avoided during therapy. By contrast, meropenem, tigecycline, and moxifloxacin had excellent activity. These results further confirm the results of previous studies regarding the susceptibility of *B. wadsworthia* [6,7,8,9,10].

## 4. Conclusions

In conclusion, *B. wadsworthia* is easily overlooked because of its slow growth and small colonies. The present case study highlights the importance of a high index of suspicion in patients with gastrointestinal tract-related cancers and/or surgical interventions and the need for early intervention with appropriate empirical and targeted antibiotic therapy covering *B. wadsworthia*.

## Data Availability

The data shown and discussed in this paper have been deposited in the NCBI GenBank with the following BioProject no.: PRJNA1131358.

## References

[1] Ligero-López J., Rubio-Mora E., Ruiz-Bastián M.D., Quiles-Melero M.I., Cacho-Calvo J., Cendejas-Bueno E. (2023). Antimicrobial susceptibility testing of anaerobic bacteria causing bacteremia: A 13-year (2010–2022) retrospective study in a tertiary hospital. Anaerobe.

[2] Cobo F., Pérez-Carrasco V., Rodríguez-Granger J., Sampedro-Martínez A., García-Salcedo J.A., Navarro-Marí J.M. (2023). Differences between bloodstream infections involving Gram-positive and Gram-negative anaerobes. Anaerobe.

[3] Zouggari Y., Lelubre C., Lali S.E., Cherifi S. (2022). Epidemiology and outcome of anaerobic bacteremia in a tertiary hospital. Eur. J. Intern. Med..

[4] Baron E.J., Curren M., Henderson G., Jousimies-Somer H., Lee K., Lechowitz K., Strong C.A., Summanen P., Tunér K., Finegold S.M. (1992). *Bilophila wadsworthia* isolates from clinical specimens. J. Clin. Microbiol..

[5] Chen X.F., Hou X., Xiao M., Zhang L., Cheng J.W., Zhou M.L., Huang J.J., Zhang J.J., Xu Y.C., Hsueh P.R. (2021). Matrix-Assisted Laser Desorption/Ionization Time of Flight Mass Spectrometry (MALDI-TOF MS) Analysis for the Identification of Pathogenic Microorganisms: A Review. Microorganisms.

[6] Bernard D., Verschraegen G., Claeys G., Lauwers S., Rosseel P. (1994). *Bilophila wadsworthia* bacteremia in a patient with gangrenous appendicitis. Clin. Infect. Dis..

[7] Urbán E., Hortobágyi A., Szentpáli K., Nagy E. (2004). Two intriguing *Bilophila wadsworthia* cases from Hungary. J. Med. Microbiol..

[8] Marina M., Ivanova K., Ficheva M., Fichev G. (1997). *Bilophila wadsworthia* in brain abscess: Case report. Anaerobe.

[9] Acker E., George M., Farooqi T., Raval M., Ramani A. (2022). Polymicrobial anaerobic sepsis due to *Bacteroides fragilis*, *Eggerthella lenta*, *Ruminoccocus gnavus*, and *Bilophila wadsworthia* in a patient with myeloproliferative neoplasm. Anaerobe.

[10] Kasten M.J., Rosenblatt J.E., Gustafson D.R. (1992). *Bilophila wadsworthia* bacteremia in two patients with hepatic abscesses. J. Clin. Microbiol..

[11] Clinical and Laboratory Standards Institute (CLSI) (2018). Performance Standards for Antimicrobial Susceptibility Testing.

[12] Kolmogorov M., Yuan J., Lin Y., Pevzner P.A. (2019). Assembly of long, error-prone reads using repeat graphs. Nat. Biotechnol..

[13] Ewels P.A., Peltzer A., Fillinger S., Patel H., Alneberg J., Wilm A., Garcia M.U., Di Tommaso P., Nahnsen S. (2020). The nf-core framework for community-curated bioinformatics pipelines. Nat. Biotechnol..

[14] Wick R.R., Holt K.E. (2022). Polypolish: Short-read polishing of long-read bacterial genome assemblies. PLoS Comput. Biol..

[15] Chen S., Zhou Y., Chen Y., Gu J. (2018). fastp: An ultra-fast all-in-one FASTQ preprocessor. Bioinformatics.

[16] Seppey M., Manni M., Zdobnov E.M. (2019). BUSCO: Assessing Genome Assembly and Annotation Completeness. Methods Mol. Biol..

[17] Nishimura O., Hara Y., Kuraku S. (2017). gVolante for standardizing completeness assessment of genome and transcriptome assemblies. Bioinformatics.

[18] Parks D.H., Imelfort M., Skennerton C.T., Hugenholtz P., Tyson G.W. (2015). CheckM: Assessing the quality of microbial genomes recovered from isolates, single cells, and metagenomes. Genome Res..

[19] Jain C., Rodriguez-R L.M., Phillippy A.M., Konstantinidis K.T., Aluru S. (2018). High throughput ANI analysis of 90K prokaryotic genomes reveals clear species boundaries. Nat. Commun..

[20] Florensa A.F., Kaas R.S., Clausen P.T.L.C., Aytan-Aktug D., Aarestrup F.M. (2022). ResFinder—An open online resource for identification of antimicrobial resistance genes in next-generation sequencing data and prediction of phenotypes from genotypes. Microb. Genom..

[21] Baron E.J., Summanen P., Downes J., Roberts M.C., Wexler H., Finegold S.M. (1989). *Bilophila wadsworthia*, gen. nov. and sp. nov., a unique gram-negative anaerobic rod recovered from appendicitis specimens and human faeces. J. Gen. Microbiol..

